# Supplementary material for: Discovery and application of insertion-deletion (INDEL) polymorphisms for QTL mapping of early life-history traits in Atlantic salmon
Source: BMC Genomics. 2010 Mar 8;11:156. doi: 10.1186/1471-2164-11-156 (PMC2838853; doi:10.1186/1471-2164-11-156)
Supplement: Additional file 2 — Information on developed 76 locus single-run INDEL panel in Atlantic salmon. Information on fluorescence labeling, primer concentrations, PCR pooling and links to alignments, INDEL motifs and GENESCAN (Burge and Karlin 1997) predictions of genes/exons are available in html format. [file 1471-2164-11-156-S2.ZIP › Additionalfile2/snpsummary3335.html]

```
Cluster 694 Contig 1

prev  Summary    Contig List  next
```

Size of Consensus sequence = 2692

Number of sequences = 37

Minimum redundancy = 6

Key

A gi|117531720|gb|EG863165.1|EG863165 EST\_ssal\_eve\_48832 ssaleve thyroid Salmo salar cDNA Salmo salar cDNA clone ssal\_eve\_566\_185\_rev 5', mRNA sequence  
B gi|24341095|gb|CA040169.1|CA040169 ssalshc503175 spleen Salmo salar cDNA, mRNA sequence  
C gi|117518101|gb|EG849860.1|EG849860 EST\_ssal\_eve\_7266 ssaleve thyroid Salmo salar cDNA Salmo salar cDNA clone ssal\_eve\_508\_328\_rev 5', mRNA sequence  
D gi|117480861|gb|EG813078.1|EG813078 EST\_ssal\_evd\_33090 ssalevd thymus Salmo salar cDNA Salmo salar cDNA clone ssal\_evd\_544\_019\_rev 5', mRNA sequence  
E gi|24392030|gb|CA061787.1|CA061787 ssalrgb512268 mixed\_tissue Salmo salar cDNA, mRNA sequence  
F gi|29314570|gb|CB503344.1|CB503344 ssalmge505294 gut Salmo salar cDNA, mRNA sequence  
G gi|117433833|gb|EG766056.1|EG766056 EST\_ssal\_evd\_42234 ssalevd thymus Salmo salar cDNA Salmo salar cDNA clone ssal\_evd\_556\_226\_rev 5', mRNA sequence  
H gi|117526734|gb|EG858461.1|EG858461 EST\_ssal\_eve\_51907 ssaleve thyroid Salmo salar cDNA Salmo salar cDNA clone ssal\_eve\_570\_270\_fwd 3', mRNA sequence  
I gi|117544075|gb|EG875520.1|EG875520 EST\_ssal\_eve\_19327 ssaleve thyroid Salmo salar cDNA Salmo salar cDNA clone ssal\_eve\_526\_119\_rev 5', mRNA sequence  
J gi|117526735|gb|EG858462.1|EG858462 EST\_ssal\_eve\_51908 ssaleve thyroid Salmo salar cDNA Salmo salar cDNA clone ssal\_eve\_570\_270\_rev 5', mRNA sequence  
K gi|117544076|gb|EG875521.1|EG875521 EST\_ssal\_eve\_19328 ssaleve thyroid Salmo salar cDNA Salmo salar cDNA clone ssal\_eve\_526\_119\_fwd 3', mRNA sequence  
L gi|117563235|gb|EG884211.1|EG884211 EST\_ssal\_evf\_42690 ssalevf mixed\_tissue Salmo salar cDNA Salmo salar cDNA clone ssal\_evf\_557\_023\_rev 5', mRNA sequence  
M gi|117530559|gb|EG862015.1|EG862015 EST\_ssal\_eve\_55105 ssaleve thyroid Salmo salar cDNA Salmo salar cDNA clone ssal\_eve\_575\_043\_rev 5', mRNA sequence  
N gi|117857207|gb|EG929903.1|EG929903 EST\_ssal\_evf\_32070 ssalevf mixed\_tissue Salmo salar cDNA Salmo salar cDNA clone ssal\_evf\_542\_233\_rev 5', mRNA sequence  
O gi|117545855|gb|EG877300.1|EG877300 EST\_ssal\_eve\_17321 ssaleve thyroid Salmo salar cDNA Salmo salar cDNA clone ssal\_eve\_523\_234\_rev 5', mRNA sequence  
P gi|117497543|gb|EG829760.1|EG829760 EST\_ssal\_eve\_42076 ssaleve thyroid Salmo salar cDNA Salmo salar cDNA clone ssal\_eve\_557\_090\_rev 5', mRNA sequence  
Q gi|117513260|gb|EG845019.1|EG845019 EST\_ssal\_eve\_37811 ssaleve thyroid Salmo salar cDNA Salmo salar cDNA clone ssal\_eve\_551\_184\_rev 5', mRNA sequence  
R gi|117552643|gb|EG883398.1|EG883398 EST\_ssal\_eve\_17015 ssaleve thyroid Salmo salar cDNA Salmo salar cDNA clone ssal\_eve\_523\_050\_rev 5', mRNA sequence  
S gi|29326979|gb|CB515753.1|CB515753 ssalrgb554129 mixed\_tissue Salmo salar cDNA, mRNA sequence  
T gi|117531709|gb|EG863154.1|EG863154 EST\_ssal\_eve\_48831 ssaleve thyroid Salmo salar cDNA Salmo salar cDNA clone ssal\_eve\_566\_185\_fwd 3', mRNA sequence  
U gi|84985658|gb|DW536008.1|DW536008 EST\_ssal\_rgb2\_427 rgb2 Salmo salar cDNA clone ssal\_rgb2\_501\_322\_fwd 3', mRNA sequence  
V gi|117459550|gb|EG791769.1|EG791769 EST\_ssal\_evd\_11911 ssalevd thymus Salmo salar cDNA Salmo salar cDNA clone ssal\_evd\_514\_304\_rev 5', mRNA sequence  
W gi|117552532|gb|EG883287.1|EG883287 EST\_ssal\_eve\_17014 ssaleve thyroid Salmo salar cDNA Salmo salar cDNA clone ssal\_eve\_523\_050\_fwd 3', mRNA sequence  
X gi|117830920|gb|EG903616.1|EG903616 EST\_ssal\_evf\_2154 ssalevf mixed\_tissue Salmo salar cDNA Salmo salar cDNA clone ssal\_evf\_501\_052\_rev 5', mRNA sequence  
Y gi|117459551|gb|EG791770.1|EG791770 EST\_ssal\_evd\_11912 ssalevd thymus Salmo salar cDNA Salmo salar cDNA clone ssal\_evd\_514\_304\_fwd 3', mRNA sequence  
Z gi|117563236|gb|EG884212.1|EG884212 EST\_ssal\_evf\_42691 ssalevf mixed\_tissue Salmo salar cDNA Salmo salar cDNA clone ssal\_evf\_557\_023\_fwd 3', mRNA sequence  
a gi|117433832|gb|EG766055.1|EG766055 EST\_ssal\_evd\_42233 ssalevd thymus Salmo salar cDNA Salmo salar cDNA clone ssal\_evd\_556\_226\_fwd 3', mRNA sequence  
b gi|117497544|gb|EG829761.1|EG829761 EST\_ssal\_eve\_42077 ssaleve thyroid Salmo salar cDNA Salmo salar cDNA clone ssal\_eve\_557\_090\_fwd 3', mRNA sequence  
c gi|117518102|gb|EG849861.1|EG849861 EST\_ssal\_eve\_7267 ssaleve thyroid Salmo salar cDNA Salmo salar cDNA clone ssal\_eve\_508\_328\_fwd 3', mRNA sequence  
d gi|117831031|gb|EG903727.1|EG903727 EST\_ssal\_evf\_2155 ssalevf mixed\_tissue Salmo salar cDNA Salmo salar cDNA clone ssal\_evf\_501\_052\_fwd 3', mRNA sequence  
e gi|117857206|gb|EG929902.1|EG929902 EST\_ssal\_evf\_32069 ssalevf mixed\_tissue Salmo salar cDNA Salmo salar cDNA clone ssal\_evf\_542\_233\_fwd 3', mRNA sequence  
f gi|117837931|gb|EG910627.1|EG910627 EST\_ssal\_evf\_12721 ssalevf mixed\_tissue Salmo salar cDNA Salmo salar cDNA clone ssal\_evf\_515\_189\_fwd 3', mRNA sequence  
g gi|84985659|gb|DW536009.1|DW536009 EST\_ssal\_rgb2\_428 rgb2 Salmo salar cDNA clone ssal\_rgb2\_501\_322\_rev 5', mRNA sequence  
h gi|117513261|gb|EG845020.1|EG845020 EST\_ssal\_eve\_37812 ssaleve thyroid Salmo salar cDNA Salmo salar cDNA clone ssal\_eve\_551\_184\_fwd 3', mRNA sequence  
i gi|89836349|gb|DY696086.1|DY696086 EST\_ssal\_rgb2\_51825 ssalrgb2 mixed\_tissue Salmo salar cDNA Salmo salar cDNA clone ssal\_rgb2\_583\_339\_rev 5', mRNA sequence  
j gi|117493329|gb|EG825546.1|EG825546 EST\_ssal\_eve\_38283 ssaleve thyroid Salmo salar cDNA Salmo salar cDNA clone ssal\_eve\_552\_046\_rev 5', mRNA sequence  
k gi|117493330|gb|EG825547.1|EG825547 EST\_ssal\_eve\_38284 ssaleve thyroid Salmo salar cDNA Salmo salar cDNA clone ssal\_eve\_552\_046\_fwd 3', mRNA sequence

2 SNPs detected

A B C D E F G H I J K L M N O P Q R S T U V W X Y Z a b c d e f g h i j k  cosegregation weighted

604 . . . - - G - - - - - - - - G G G G - - - G G - G - - G - - - - - . . . .   1/2 40.54
605 . . . - - A - - - - - - - - A A A A - - - A A - A - - A A - - - - . . . .   1/2 40.54
